# Novel Machine Learning-Based Method for Estimation of the Surface Area of Porous Silica Particles

**DOI:** 10.1021/acs.iecr.3c02785

**Published:** 2023-10-27

**Authors:** Roja P. Moghadam, Chinmay A. Shukla, Vivek V. Ranade

**Affiliations:** Multiphase Reactors and Process Intensification Group, Bernal Institute, University of Limerick, Limerick V94T9PX, Ireland

## Abstract

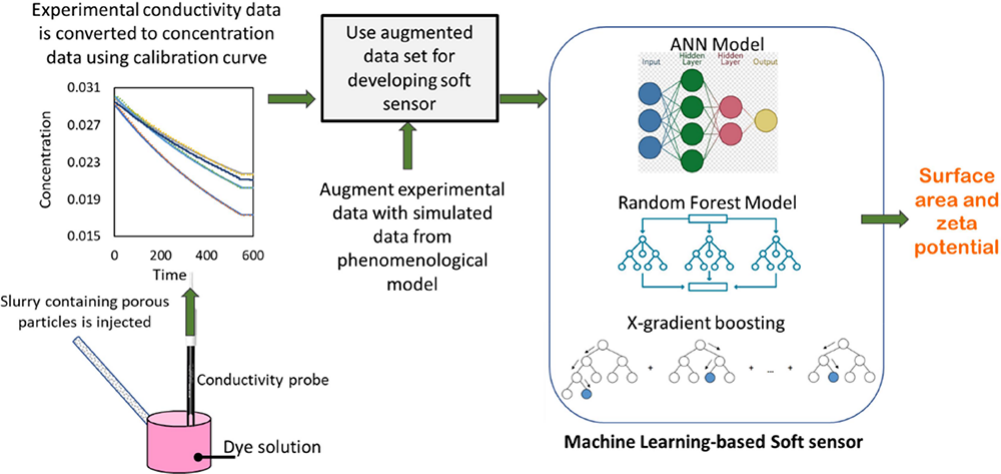

This work reports a novel and quick method to estimate the surface area of porous materials. Conventionally, surface area measurement requires the BET method/N_2_ adsorption experiment which is time-consuming. In this work, we developed a method based on machine learning (ML) and the adsorption of a conductive dye on porous materials. The rate and quantity of dye adsorption, which is characterized by dynamic measurement of conductivity, provide an indirect measure of surface area and zeta potential. An ML-based soft sensor is developed to relate the measured conductivity profiles with surface area and zeta potential. A phenomenological model on dye adsorption is also developed, validated, and used to augment experimental data for training the soft sensor. The developed method was tested for porous silica particles with a range of surface areas (250–1100 m^2^/g) and zeta potential (−17 mV: −29 mV). The developed soft sensor was able to estimate the surface area and zeta potential quite well. The developed approach and method reduce overall measurement time for surface area from several hours to a few minutes. The method can potentially be implemented in continuous plants producing porous materials like silica.

## 1 Introduction

Porous materials are used in a wide range of engineering applications including catalysis, adsorbents, energy storage, biomedical, and drug delivery platforms. They offer a favorable combination of properties, including structural, mechanical, and thermal characteristics.^1^ Porous materials are categorized broadly into three types (microporous, mesoporous, and microporous) based on the pore size (and distribution). For a given type of porous material, porosity, surface area per unit weight, and surface characteristics essentially determine their performance.^2−4^ The surface area and surface charge are two of the most significant surface characteristics that play a crucial role in processes such as adsorption, conductivity, and drug delivery systems as pharmacokinetic properties. Surface charge is typically characterized by measuring zeta potential which is an index of the degree of attraction between particles in a liquid suspension.^5,6^ Zeta potential measurements are also employed to assess the formation and relative distribution of species, determine the apparent surface coverage, evaluate adsorption and reactivity, and devise synthesis strategies to attain desired properties.^7,8^

Considering the central role of the surface area and the zeta potential in determining the functional performance of porous materials, accurate measurement of these two quantities is essential. Zeta potentials cannot be measured directly. Instead, electrophoretic mobility of particles is measured, and the Gouy–Chapman theory is used to obtain the value of zeta potential from these measurements.^9^ Surface area is usually measured by the Brunauer–Emmett–Teller (BET) theory. In this method, nitrogen adsorption isotherm is measured at liquid nitrogen temperature, 77 K, and the BET and BJH theory is used to estimate the total specific surface area, pore volume, and pore volume distribution. For these adsorption isotherm measurements, it is necessary to use dry and degassed samples.^10−13^ The time required for the entire adsorption experiment is dependent on the number of data points requested in the isotherm. A single point BET gives a quick estimate; however, it is not accurate, and hence, full BET or entire adsorption isotherm is generally collected, which requires significant time. These methods of measuring surface area and zeta potential are off-line and time-consuming, which make them not very useful for online quality checks of porous materials produced via a continuous process. In our research group, we are developing a continuous process for manufacturing porous silica particles.^14^ The goal is to develop a modular table-top process to manufacture silica particles of the desired surface area and zeta potential. It is therefore essential to develop a quick method to estimate the surface area and zeta potential that can be implemented for online analysis (or at least quick like online analysis) of produced silica particles. That is the focus of this work.

**Figure 1:**
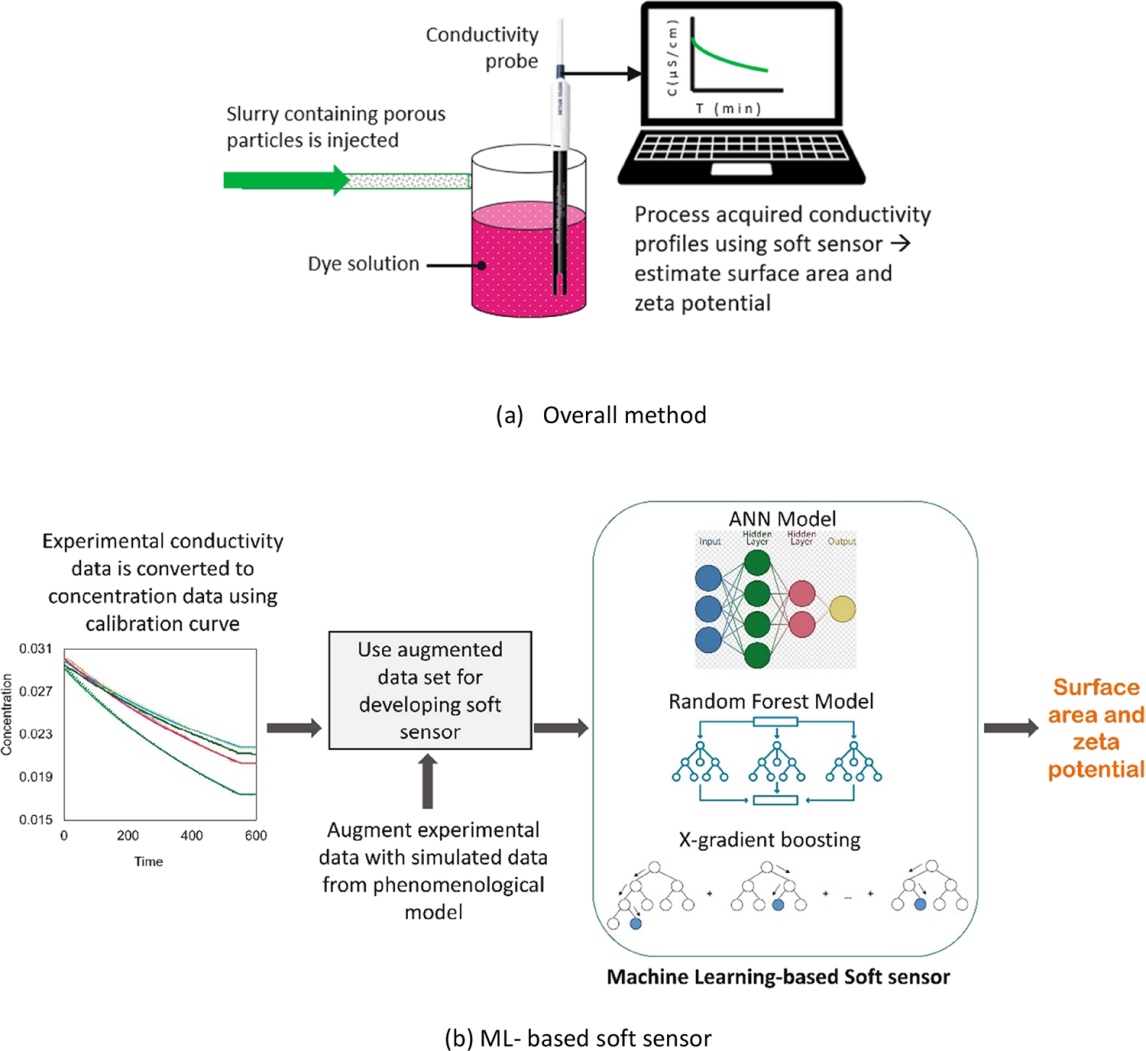
Schematic of the developed quick method for estimating the surface area and zeta potential of porous materials: (a) overall method and (b) soft sensor.

In this work, we present a novel and quick method for estimating the surface area and zeta potential. The key concept underlying the proposed quick method is to estimate the surface area and zeta potential of porous materials by using the dynamics of dye adsorption on these materials. The rate and extent of dye adsorption on porous materials depend on the surface area and zeta potential.^15^ Dynamics of dye adsorption may be quantitatively characterized using simple measurement techniques like turbidity measurements or conductivity measurements.^16,17^ In the present work, we use the adsorption of a conductive dye on porous materials and characterization of dynamics using conductivity measurements. Appropriate dye (cationic or anionic) may be selected based on the nature of porous materials being investigated. The proposed method is shown schematically in Figure 1. Figure 1a illustrates the overall process of dye adsorption experiments and conductivity measurements. The slurry containing porous particles is injected in an off-line manner or can be directly connected to a sampling port of a continuous process stream. The dynamic measurements of conductivity are then used with ML-based soft sensors to estimate the surface area and zeta potential. A schematic process that explains the concept of the soft sensor for relating conductivity profiles to the surface area and zeta potential is shown in Figure 1b.

The method relies on dye adsorption measurements, which can be completed in a short time. We developed and tested this new method for porous silica particles with a range of surface areas (250–1100 m^2^/g) and zeta potential (−17 mV: −29 mV). Experimental setup and procedures are discussed in Section 2. We have also developed, validated, and used a phenomenological model to generate simulated results to augment the experimental data set. Machine learning (ML formalisms, namely, artificial neural network (ANN), random forest (RF), and Extreme Gradient Boosting (XGBoost)) were used to test the prediction performance for the surface area and zeta potential. Subsequently, we introduced a relationship between the surface area and zeta potential, enabling us to predict the zeta potential. The phenomoenological model and development of the soft sensor are discussed in Section 3. The results of the application of the developed method to porous silica particles through two scenarios are discussed in Section 4. Although the application of the method is illustrated here with silica particles, the developed method is quite general and can be applied to many other porous materials than silica. The method offers a much quicker estimation of surface area and zeta potential than conventional measurement techniques (in a few minutes instead of several hours) and will be useful for researchers and industries interested in characterizing surface area and zeta potential. The method is also amenable for implementation in continuous processes that produce porous materials.

## 2 Experimental Section

**Figure 2:**
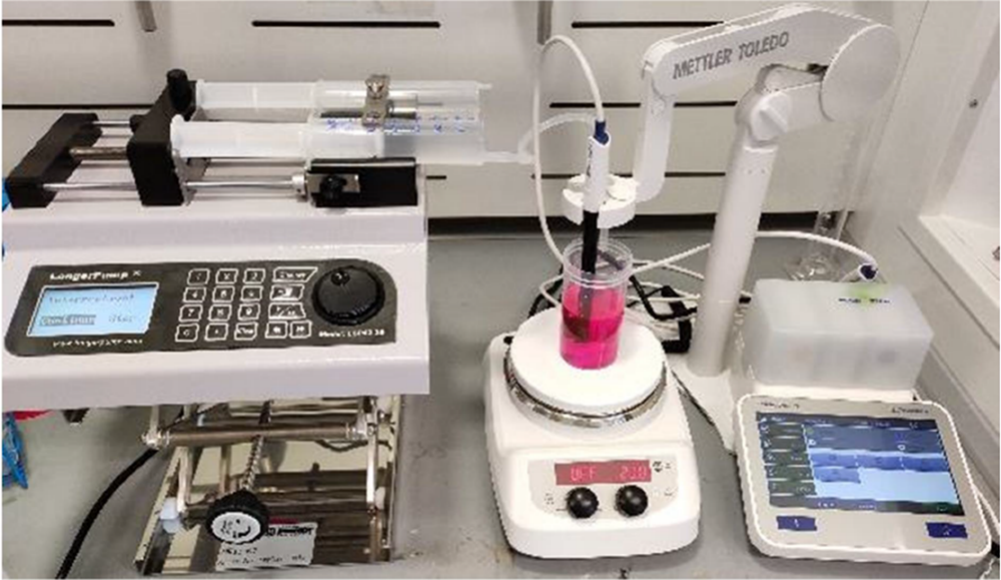
Experimental setup for dye adsorption.

The experimental setup used for dye adsorption experiments carried out in this work is shown in Figure 2. It comprises a beaker containing a dye solution, a magnetic stirrer, a conductivity probe, and a syringe pump for injecting a slurry of porous particles. The magnetic stirrer was used to ensure good mixing during the adsorption process. A stirring speed of 500 rpm was found to be suitable to achieve good mixing (mixing time of less than a second) without causing a vortex formation. Conductivity measurements were carried out using a METTLER TOLEDO SevenExcellenceTM S470 pH/conductivity benchtop meter. An LSP02-2B dual channel syringe pump with a syringe volume of 60 mL was used for injecting a slurry of porous particles.

In this work, we developed and tested the new method of estimating the surface area and zeta potential for porous silica particles. We obtained samples of commercial silica particles for this purpose. Considering the range of zeta potential (−17 mV: −29 mV) of these particles, a cationic dye was selected. In this work, we used Rhodamine B as a water-soluble cationic dye. Rhodamine B was previously reported to undergo rapid adsorption (fast adsorption kinetics) on mesoporous silicas.^18^ Rapid dye adsorption is essential for developing a quick method for surface area estimation. Among other cationic dyes reported in previous studies, methyl violet and basic fuchsin were avoided as they were relatively more toxic (category 2 carcinogen). The malachite green dye is both carcinogenic and genotoxic (reproductive toxicity: category 2). The easy availability and relatively benign nature of Rhodamine B encouraged us to select it for our proof-of-concept studies. The developed approach and method are quite general and can be applied for any quick adsorbing dye. The molecular size of Rhodamine B is approximately 1.5 nm.^19^ The small size of Rhodamine B molecules compared to the pores of silica particles considered in this work (see measured pore size distributions in Figure S5 of Supporting Information) allows it to quantify full surface area and therefore make it suitable for characterizing surface area via adsorption. A Rhodamine B (ThermoFisher, purity> 95 wt %) stock solution of 1 mg/mL concentration was prepared by dissolving a specific amount of powdered rhodamine into deionized water (DI) using a magnetic stirrer. This concentration of dye was selected based on the conductivity probe’s range (0.01–1000 mS/cm). The dye stock solution quantity (3 mL) was selected such that the final conductivity after completion of dye adsorption was more than the lowest measurable value by the probe.

To start the experiment, 100 mL of DI water was taken in a beaker, and the initial conductivity was measured. Then 3 mL of dye stock solution was added to DI water. As the dye stock was added to DI, conductivity increased. The conductivity value of the dye solution was measured after achieving a stable reading (∼10 s). Then 40 mL of slurry containing 0.1 wt % of silica particles was added using a syringe pump. The 50 mL silica suspension was sonicated before being filled into a plastic syringe. The silica slurry was dosed at 5 mL/min by using a syringe pump for 480 s. A higher addition rate or one-shot addition causes rapid conductivity change and may also lead to the potential influence of mixing and sensor dynamics on measured conductivity profiles. As the silica slurry is added to the dye solution, the dye in the solution starts adsorbing the porous silica particles. The dynamics of adsorption depend on the surface area and silica-dye interactions. Adsorption causes a reduction in the dye concentration, which is reflected in the measured conductivity of the solution. The conductivity of the dye solution was continuously monitored and measured every second. The concentration of dye was calculated from the conductivity using the calibration curve (Figure S1 of Supporting Information). These experiments were repeated three times, and error bars for conductivity profiles of various silica particles via experimental repetition were provided in Figure S2 of Supporting Information.

For zeta potential measurement, all silica slurries were prepared with a concentration of 1.66 mg/mL and pH ∼ 6–6.5 (pH of silica slurry with DI water) and were sonicated to obtain a uniform suspension. Preliminary experiments carried out with different concentrations of silica particles indicated that the observed standard deviation of measured zeta potential is lowest at the concentration of 1.66 mg/mL (see Figure S3 of Supporting Information). The measurement was done by Malvern Zetasizer Nano ZSP. The surface area measurements were carried out using Quantachrome Autosorb AS-1 nitrogen adsorption equipment. Around 100 mg of each silica sample was placed in a sample cell and was degassed for 17 h under 120 °C before carrying out N_2_ adsorption. Surface area values were in agreement with reported values in the previous literature.^20−22^ Typical plots of isotherm adsorption and desorption of N_2_ for silica samples are presented in Figure S4 of Supporting Information.

The measured values of surface area and zeta potential for four commercial silica samples investigated in this work are listed in Table 1 (first three columns). It can be seen that the measured surface area values are in the range of 249 to 1082 m^2^/g (first four rows). However, there was a lack of samples in the range of surface area 514–1082 m^2^/g. Therefore, mixed samples were prepared with different mass fractions (0.2, 0.5, and 0.8) of SLC500 and MCM41. Calculation of surface area for a mix of particles in different sizes is described in Section 5 of Supporting Information. These mixtures resulted in surface area values in the desired range. The zeta potential measurements for all samples were repeated for three times to estimate error bars on the reported value.

**Table 1:** Comparison between Experimentally Measured Characteristics of Commercial Silica Particles and Modelling Data

sample name	zeta potential (mV)	surface area (m^2^/g)		P1 (m^3^/g)	pore diameter (nm)
		experiment	model		
Merk (SLC 500)	–20.58 ± 0.36	514	515	12.5	6.2
Glantreo (XDP3050)	–21.78 ± 0.35	287	289	25	19
Glantreo (MCM41)	–28.66 ± 0.18	1082	1090	115	3
Glantreo (SL197SC)	–17.71 ± 0.20	249	250	27	5
Mix28 (SLC500:MCM41::0.2:0.8)	–27.1 ± 0.55	968	977	70	3–6.2
Mix55 (SLC500:MCM41::0.5:0.5)	–25.93 ± 0.24	798	795	38	3–6.2
Mix82 (SLC500:MCM41::0.8:0.2)	–23.63 ± 0.34	628	625	20	3–6.2

## 3 ML-Based Soft Sensor for Estimating the Surface Area and Zeta Potential

The key element of the proposed quick method is to develop an ML-based soft sensor for relating measured conductivity profiles to surface area and zeta potential. If an appropriate soft sensor can be developed to relate measured conductivity profiles to surface area and zeta potential, it will reduce overall measurement time from several hours to a few minutes. The development of ML-based soft sensors, however, requires large data sets. Considering the time-consuming nature of BET measurements, a rather limited number of measurements were available for training the soft sensor. In order to augment the training data set, we developed a phenomenological model for simulating measured conductivity profiles. The model was verified by comparing the simulated results with the experimentally measured conductivity profiles. The validated model was then used for generating simulated results that were included in the training data set. The development and verification of the phenomenological model are discussed in Section 3.1. In order to reduce the burden on training, the potential relationship between surface area and zeta potential for considered silica particles. This is discussed in Section 3.2. Using the results of experiments and model, the development of the ML-based soft sensor is discussed in Section 3.3.

### 3.1 Phenomenological Model To Simulate Dye Adsorption on Silica

Dye adsorption experiments are assumed to be isothermal. Since a slurry containing silica particles is added to the dye solution, the volume of the solution varies with time. The overall process may be described by following mass balances:^23,24^
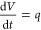
1
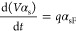
2
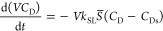
3where *V* is the volume of solution, *t* is the time, *q* is the flow rate of slurry, α_s_ is the volume fraction of solids, *C*_D_ is the concentration of dye, *k*_SL_ is the solid–liquid mass transfer coefficient, and *S̅* is the surface area per volume. α_sF_ is the volume fraction of solids in fed slurry and *C*_Ds_ is the equilibrium concentration of dye on solid surface. Initial and boundary conditions may be written as
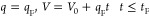
4
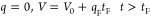
5
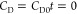


Thus, we get
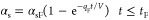
6
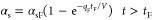
7

The equilibrium concentration of dye on solid surface may be related to the mass fraction of dye adsorbed on the solid (*y*_Ds_) using the Langmuir isotherm as
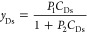
8where *P*_1_ and *P*_2_ are parameters of the adsorption isotherm.

The overall mass balance may be written as
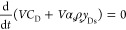
9

We thus get
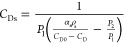
10

For a low concentration of dye, *P*_2_ → 0:
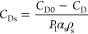
11

The dye mass balance, Equation 3 may therefore be rewritten as
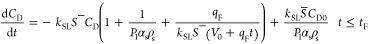
12where , and *m*_f_ and *s* are mass of solid in slurry and surface area, respectively.

The solution of model equations using “dsolve” as a solver of MATLAB (R2022a) gives
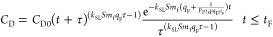
13
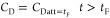
14where .

At *t* = *t*_F_, the slurry injection was stopped. More details of adsorption model are discussed in Section 7 of Supporting Information.

Experimentally measured conductivity profiles were converted to concentration profiles by using the calibration curve. Since for all dye adsorption experiments were carried out with same stirring speed and solid loading, the solid–liquid mass transfer coefficient, *k*_Sl_, was considered to be the same for all experiments. The value mass transfer coefficient was estimated using the diffusivity, and the Sherwood number^25^ was 10^–4^ m/s and was fixed for all cases. The values of parameters *P*_1_ and *S* were fitted by minimizing the mean absolute error between the predicted and experimental concentrations. The grid search technique was used for this purpose. This technique is a hyperparameter optimization function that explores various combinations of hyperparameters within the specified search space. It is particularly effective when dealing with a limited number of hyperparameters and a predetermined set of possible values for each.^26^

**Figure 3:**
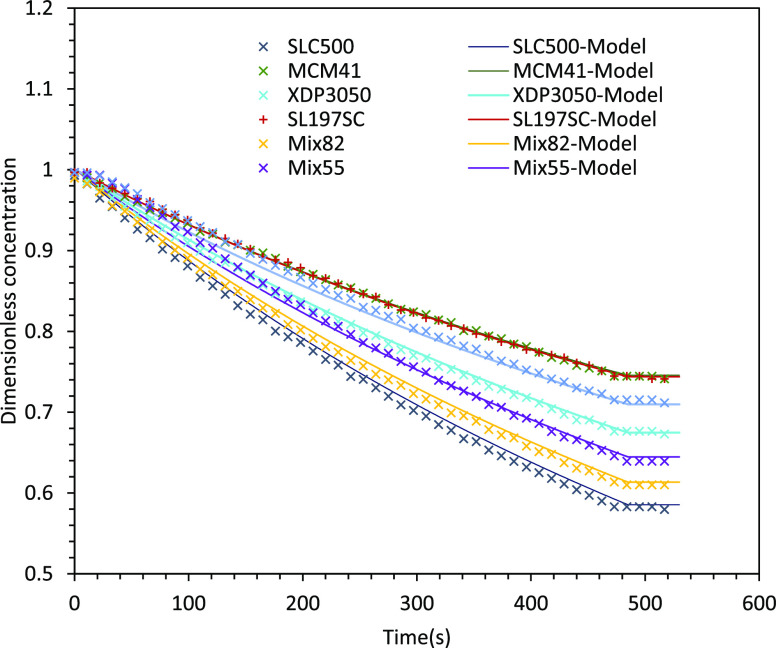
Normalized dye dimensionless concentration profiles: Experimental (symbols) and simulated (continuous lines).

The comparison of simulated and experimental dye concentration profiles for different silica particles is shown in Figure 3. Though data was recorded at every second, for facilitating the visualization, the experimental results were plotted at intervals of 10 s. It can be seen that the developed model was able to describe the experimental profiles reasonably well. The values of fitted parameters and comparison of experimental and fitted surface areas are listed in Table 1. It is important to note that cationic dye molecules carry charged or polar functional groups. When a dye molecule with a net charge approaches the silica surface, electrostatic interactions occur between the charged groups on the dye and the oppositely charged ones on the silica. These interactions can result in adsorption of the dye onto the silica surface.^27^ van der Waals forces, including London dispersion forces, can contribute to the adsorption process. These forces arise due to temporary fluctuations in electron distribution, leading to attractive interactions between nonpolar regions of the dye molecules and the silica surface. In cases where silica has a porous structure, dye molecules may enter the pores and become physically trapped within the pores. This process, known as pore filling, can increase the overall adsorption capacity of silica for dyes.^28^ The rate of dye adsorption on silica depends on the dye concentration, total surface area/porosity, surface charge/zeta potential of silica, pH of the solution, silica functionalization, and temperature. Thus, silica samples with different total surface areas/porosity or zeta potentials give different concentration profiles of dye. According to Figure 3, it can be seen that dye adsorption by SLC500 silica is greater than that in other samples.

**Figure 4:**
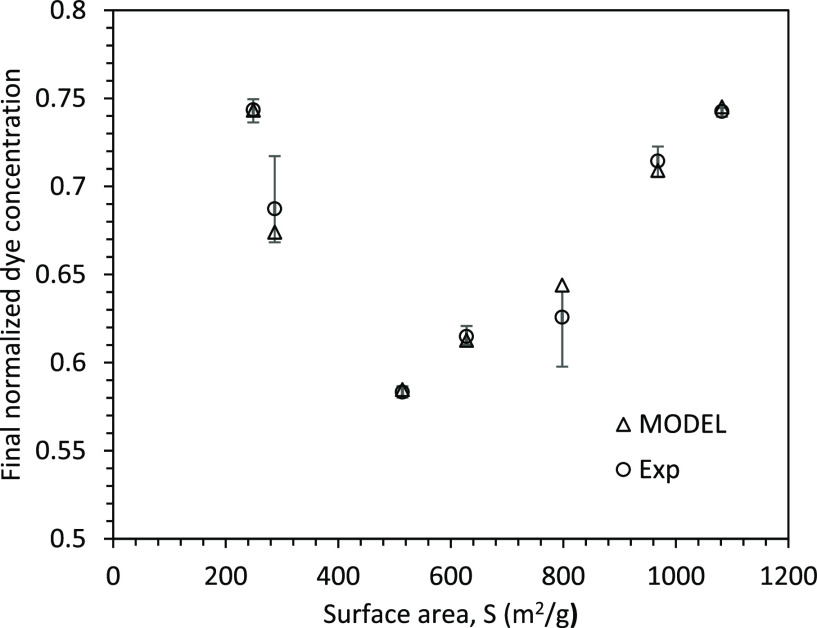
Final normalized dye concentration versus surface area.

Furthermore, there is a significant difference between these silica samples that can be attributed to their total surface areas and/or zeta potential/surface charge and/or silica functionalization (dye-silica interaction). The comparison of final dye concentration between experimental and modeling for different silica is shown in Figure 4. It can be seen that the simulated results agree very well with the experimental data. It is interesting to note that the final dye concentration exhibits minima with respect to the surface area, which looks counterintuitive at the first look. The observed nonmonotonic nature of variation of final dye concentration with respect to surface area is most likely because of the influence of zeta potential or surface charges. This prompted us to examine such a potential relationship between zeta potential and surface area, which is discussed in the following section. These data exhibit a U-shaped trend, and the minimum point is related to silica with a surface area of ∼500 m^2^/g.

### 3.2 Relationship between Zeta Potential and Surface Area

**Figure 5:**
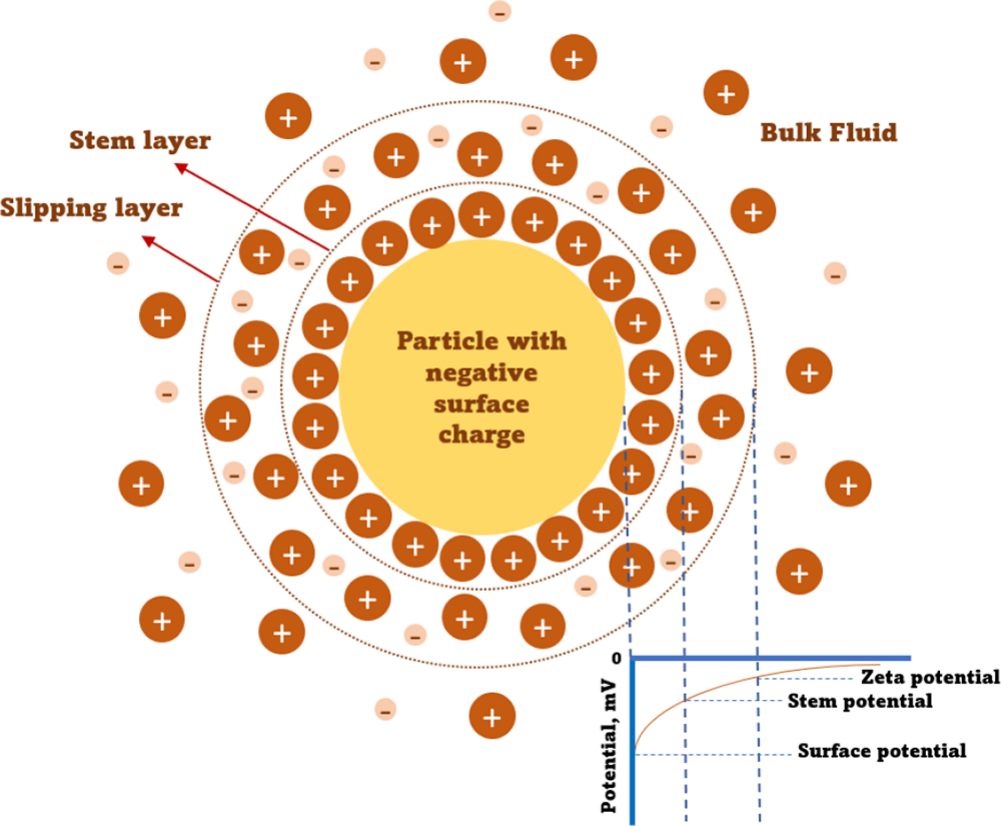
A schematic showing the electric double layer and zeta potential.

The theory of an electric double layer (EDL) was established decades ago to describe the distribution of ions and the electrostatic potential surrounding a charged particle. The term “double layer” pertains to the two layers of charges, one being the fixed charges on the particle surface and the other consisting of excess counterions in the aqueous solution. The zeta potential is the difference between the potential value at the bulk of base fluid and the potential value at the stationary layer of fluid around the particles (see Figure 5).

The Poisson equation, which describes the electrostatic attraction or repulsion of counterions and co-ions to the surface, and Boltzmann’s relation, which characterizes the diffusion of ions due to thermal motion, are essential components of the EDL theory. This theory is useful to find a relation between the surface charge density and the zeta potential. The surface charge density (σ) is calculated as^9^
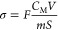
15where *C*_M_ is the acidic or basic reagent concentration, *V* is the reagent volume, *m* is the weight of powder, and *S* is the specific surface area.

The Gouy–Chapman theory establishes a direct relationship between zeta potential and the “effective charge density” based on the sum of charge density on a flat surface and the surrounding ions enclosed by the slipping plane.^9^ The resulting nonlinear Poisson–Boltzmann equation has an exact solution in the 1-dimensional case, which is known as the Gouy–Chapman equation for a flat-charged surface:
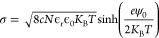
16where *c* stands for the ion concentration, *N* is the Avogadro constant, *k*_B_ is Boltzmann’s constant, ϵ_r_ is the relative permittivity or dielectric constant, ϵ_0_ is the permittivity of vacuum or free space, *T* is the temperature, and σ and ψ_0_ are the surface charge density and surface electrostatic potential, respectively. While the Gouy–Chapman equation describes the relation between ψ_0_ and σ, the very same relation exists between zeta potential (ξ) and the effective charge density (σ_eff_) described earlier:^29^
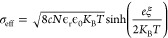
17

Based on the above three equations, surface area may be related to the zeta potential as
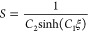
18

Considering the positive values of surface area and negative values of zeta potential and by applying the mapping method, eq 18 can be presented by the following form (more details in Section 8 of the Supporting Information):
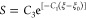
19

Therefore, the zeta potential versus surface area will be based on the natural log function. But for this case, for small values of *C*_1_(ξ – ξ_0_), eq 19 with some rearrangement can be written in a linear form as
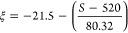
20

**Figure 6:**
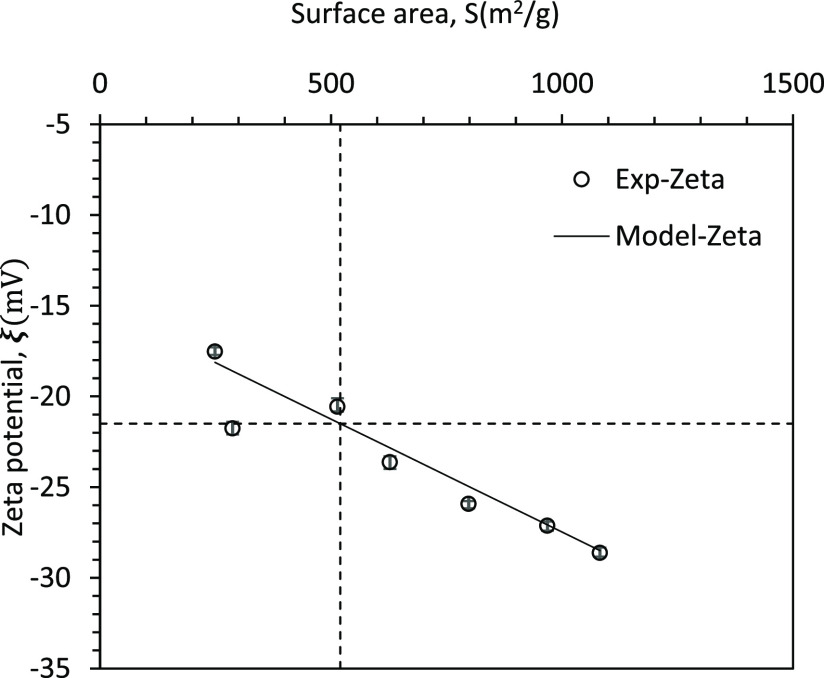
Relation between zeta potential and surface area.

The measured values of zeta potential and surface area of seven samples are shown in Table 1 and a line corresponding to eq 20 is shown in Figure 6. It can be seen that eq 20 represents the data reasonably well.

### 3.3 Development of the Machine Learning-Based Soft Sensor

As discussed in Section 1 and shown schematically in Figure 1, we were initially hoping to use the measured and simulated conductivity profiles to develop a machine learning (ML)-based soft sensor for estimating zeta potential and surface area. However, after establishing the relationship between zeta potential and surface area (eq 22/ Figure 6), it may not be necessary to develop a soft sensor for estimating both of these characteristics. We therefore decided to develop a soft sensor for estimating surface area based on the measured conductivity profile. For developing the soft sensor, we also used simulated conductivity profiles obtained from the validated phenomenological model (as discussed in Section 3.1).

**Figure 7:**
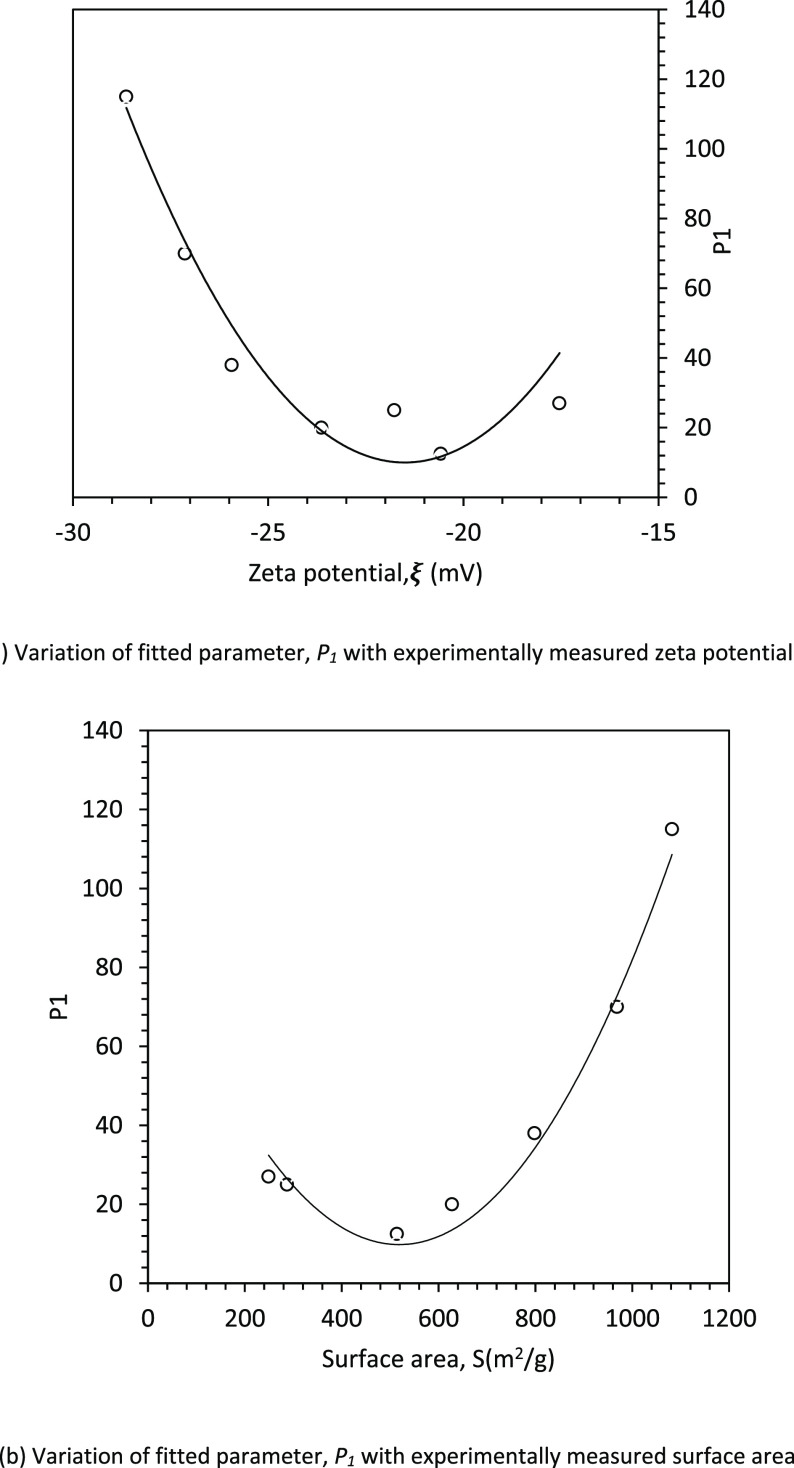
Parabolic relationship of *P*_1_ with zeta potential and surface area.

The relationship between the zeta potential and surface area also had an interesting consequence. Close examination of fitted adsorption parameter *P*_1_ indicates the parabolic type of relationship with zeta potential (see Figure 7a) and therefore with surface area as well (see Figure 7b). The continuous lines seen in Figure 7 are based on the following equations:
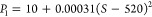
21
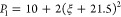
22

It can be seen from Figure 7a,b and eq 21 and 22 that there is a possibility of having two values of surface area for a given value of *P*_1_. This leads to similar conductivity profiles (with one value of *P*_1_) for two values of surface area equidistant from 520 m^2^/g (the vertex of the parabola seen in Figure 7b). Considering the similar conductivity profiles on both sides of the vertex, we developed two different soft sensors by dividing the data (both experimental and simulated) into two parts:

Soft sensor 1 (SS1): data with surface area <520 m^2^/g

Soft sensor 2 (SS2): data with surface area >520 m^2^/g

We developed various types of soft sensors by modifying the input and output variables and fine-tuning the hyperparameters. For developing the SS1, three experimental and 51 simulated concentration profiles were used (a total of 54 data sets). Preliminary numerical experiments indicate that a few points at key locations from the entire conductivity profiles are adequate for capturing the relevant information. Based on these numerical experiments, four points of each conductivity profile (at 200, 300, 400, and 485 s) were used for developing the soft sensors. The points close to the start were not found to be suitable due to higher fluctuations (in the experimental profiles). The latter part of the profile was found to be sensitive to relevant characteristics and, therefore, was used. The final point (stopping the pump), representing the final dye concentration, was found to be very important. Therefore, the data set included four input variables (with a relatively uniform distance from the middle to the end of the profiles) and one output with 54 records. A higher number of points did not result in a significant change in model output, whereas it led to an increase in the number of input variables, and with a lower number of points, the accuracy of the model decreased.

For example, the accuracy of the soft sensor model with one point (at 200 s) as input decreased by 0.2%. Furthermore, employing a profile as an input variable offers a more reliable technique compared with using a single point. This is because profiles often exhibit significant variations in slope, and these differences can be effectively leveraged by the soft sensor for accurate detection and estimation purposes. The normalized data were applied to all models. The surface area was used as the output variable for the model development and training. Considering the relationship between the zeta potential and surface area (Section 3.2), the zeta potential was not separately considered as output. Zeta potential was estimated from the surface area obtained from the soft sensor and eq 20. Three machine learning formalisms, namely, artificial neural networks (ANN), random forest (RF), and X-gradient boosting (XGB) were used for developing SS1. The details of these three formalisms and their application for the present case are briefly discussed in the following subsections.

The methodology that was used for developing SS1 was also used for developing SS2 using the data beyond the vertex (surface areas >520 m^2^/g). Four experimental and 109 simulated profiles were used. The performance of these two soft sensors is discussed in Section 4. Considering that these two soft sensors are needed to simulate samples with surface areas in the range from less than 520 m^2^/g and more than 520 m^2^/g, an attempt was made to develop a third soft sensor, SS3 with applicability over an entire range of surface areas considered in this work (from 250 to 1100 m^2^/g). In order to achieve this, it is essential to provide additional information to avoid the difficulties arising from the parabolic nature shown in Figure 7b. We therefore used zeta potential as an additional input parameter to predict the surface area through the soft sensor. This additional information about zeta potential allows the soft sensor to distinguish similar conductivity profiles obtained at two different values of surface area. The development of SS3 was carried out using the data set of five input variables and one output with 7 experimental and 160 simulated conductivity profiles. In all soft sensor models, the natural logarithm of the surface area was used as the output variable, which was subsequently transformed using the exponential function to obtain the final output. This work was conducted on the Python 3.7 platform, using the sklearn and xgboost libraries^30,31^ and by randomly splitting the data set into train (70%), validation (15%), and test (15%) data. These libraries provide some prebuilt packages and algorithms to build some ML models. The developed and tuned ML models were evaluated on extrapolation performance by using some unseen data outside of the training range to ensure robustness. The performance of the models was calculated using the following equations:
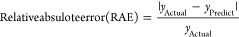
23
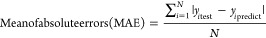
24

#### 3.3.1 ANN Models

**Figure 8:**
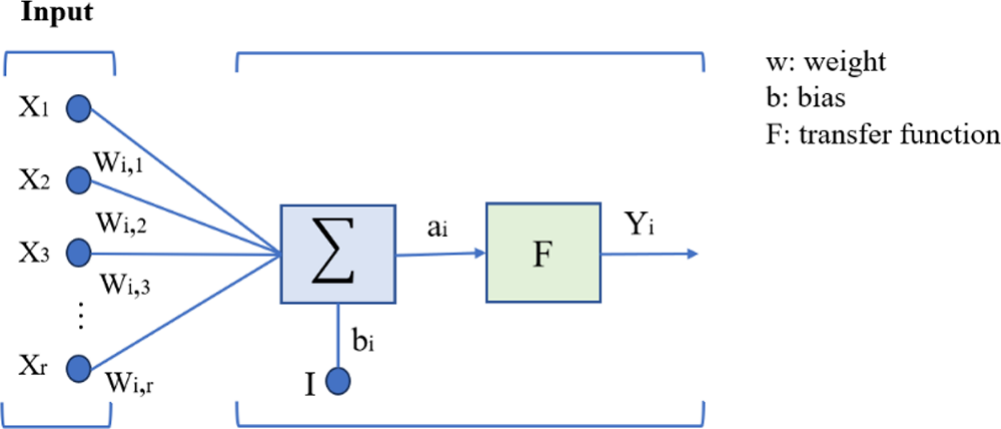
Schematic structure of artificial neuron.

The number of hidden layers and their neurons and their interconnections are important factors in the ANN structure. A schematic structure of the artificial neuron is presented in Figure 8. Using too few neurons in the hidden layers can result in underfitting, and applying too many neurons can cause overfitting and increase in time needed for training. A single hidden layer can be applied for any function with a continuous structure from one finite space to another.^32^ Therefore, the first structure of the ANN model in this work was started with one hidden layer, and its performance was compared with multiple hidden layers models. There are some empirically derived rules of thumb to determine the number of layers and their size. Based on the most common rule, the optimal size of the hidden layer is usually between the size of the input and the size of the output layers. There is another thumb rule that helps with supervised learning problems and usually prevents overfitting^32^ is
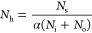
25Whereas *N*_s_, *N*_i_, and *N*_o_ are the number of samples in the training data set, the number of input neurons, and the number of output neurons, respectively. An arbitrary scaling factor, α is typically within the range of 2–10 that 2 often works without overfitting. In the first scenario (surface areas <520 m^2^/g), the input layer contained 4 neurons and the output layer contained 1 neuron. Using the aforementioned equation and *N*_s_ = 38, the maximum number of neurons in the hidden layer was calculated to be 3. After some preliminary numerical tests with the hyperbolic tan function (tanh), the final structure of the ANN model was selected with one hidden layer including 2 neurons, with a total number of parameters 13. For the second range (surface areas >520 m^2^/g) and *N*_s_ = 79, one hidden layer with 5 neurons showed the best performance. For SS3, corresponding to the entire data, *N*_s_ = 117, the maximum number of neurons in the hidden layers was found to be 9. Through a preliminary assessment of various architectures, a two-layer configuration with (5,3) neurons was chosen.

In this study, the Limited-memory Broyden–Friedrich–Goldfarb–Shanno algorithm (L-BFGS) as an optimizer in the family of quasi-Newton methods was used to iteratively update network weights based on the training data. The maximum number of iterations in this solver is determined by the number of epochs, which is typically a large value of hundreds or thousands to allow the learning algorithm to continue until the error from the model has been sufficiently minimized. The random state was employed to determine random number generation for weight and bias initialization as well as the train-test split. The optimum hyperparameters including initial learning rate (step size or the proportion that weights are updated= 0.0019), maximum number of iterations (=100), were achieved by grid search as a tuning technique.

#### 3.3.2 Random Forest Model

**Figure 9:**
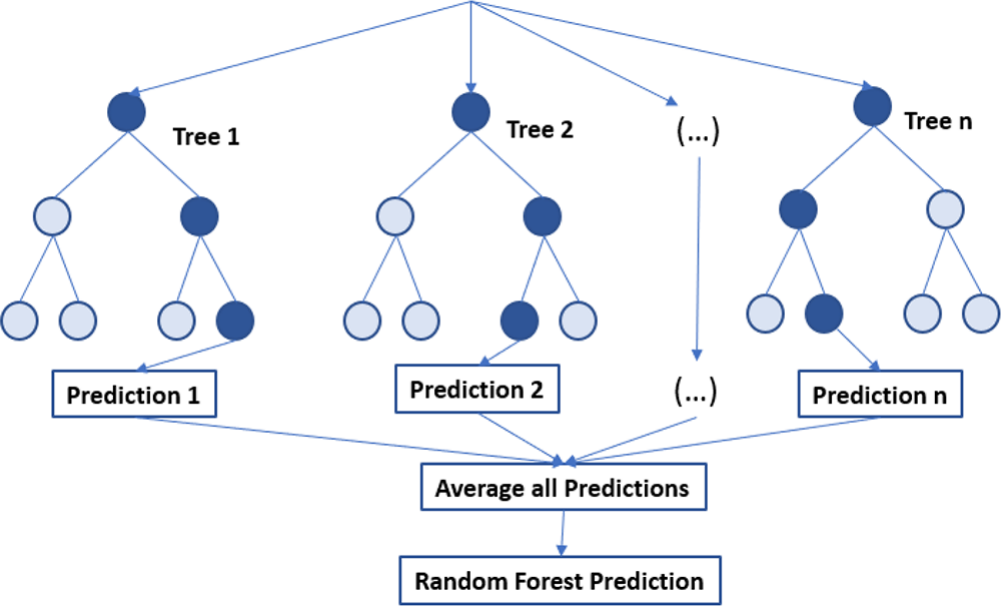
Process of random forest model.

The random forest regression algorithm is a type of supervised learning that applies ensemble learning techniques for regression. This model uses the bootstrap method to combine multiple machine learning algorithms and generate predictions more accurate than those of a single model. During training, the algorithm constructs numerous decision trees and calculates their predictions in parallel. The bootstrap technique randomly selects a subset of data from the training set without replacement, reducing bias and variance within a noisy data set. The simplest RF model with random features selects a small group of input variables on which to split at each node. RF has several advantages, including its ability to prevent overfitting by aggregating multiple trees and its increased robustness compared to decision trees.^33,34^ The process of the RF algorithm is presented in Figure 9. This model was implemented by RF regression algorithm and based on sklearn library in python with minimum samples split 2. The best model with optimum hyper parameters including number of estimators (=200), maximum depth (=5), minimum sample of leaf and maximum features (=4) was selected by grid search technique. The SS3 was tuned at the number of estimators of 1000 and maximum depth of 6.

#### 3.3.3 Extreme Gradient Boosting Model

**Figure 10:**
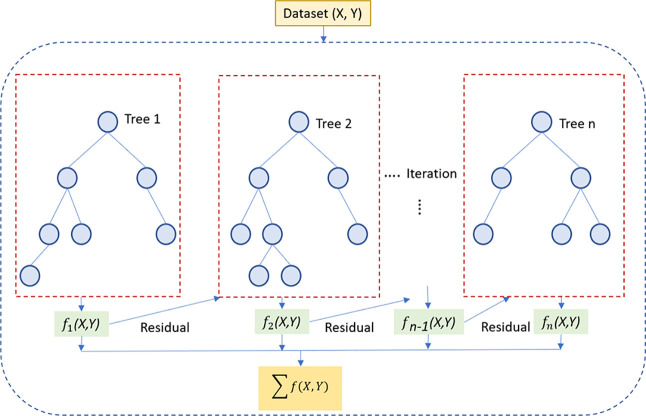
Structure of the XG-Boost model.

Extreme gradient boosting is an extension to gradient boosted decision trees (GBM) and is specially designed to improve speed and performance. Gradient boosting is one of the most powerful techniques for building predictive models and to minimize the loss function by adding weak learners by using a gradient descent optimization algorithm. XG-Boost algorithm is a sequential model in which each subsequent tree is dependent on the outcome of the last. According to Figure 10, the overall ensemble in this architecture unlike the RF algorithm does not work in parallel and during each iteration, and the previous predictors are corrected with residuals.^35^ This model was implemented by XG boosting regression algorithm and based on the XG-Boost library. The best model for SS1 and SS2 with optimum hyperparameters including number of estimators (number of gradient boosted trees = 1500), subsample (the fraction of samples to be used when constructing each tree = 0.4), maximum tree depth (=4), and learning rate (=0.01) was selected by k-fold cross validation technique. These hyper parameters for SS3 were considered as 6000, 0.1, 2, and 0.009, respectively.

Although the approach of these three machine learning based models is to predict the target value by minimizing estimation errors, there are notable differences among them. These differences encompass the selection process for subsets of features within each tree, the time required for training, and the types of hyperparameters utilized.

## 4 Performance Evaluation of Developed Soft Sensors

The evaluation of soft sensor models’ performance was done based on commonly used performance indices and by considering some unseen data outside of the training range. Some initial values in surface area (for example, SL197SC silica with a surface area of 249 m^2^/g) were considered unseen data in the test and were calculated as an extrapolated point.

**Figure 11:**
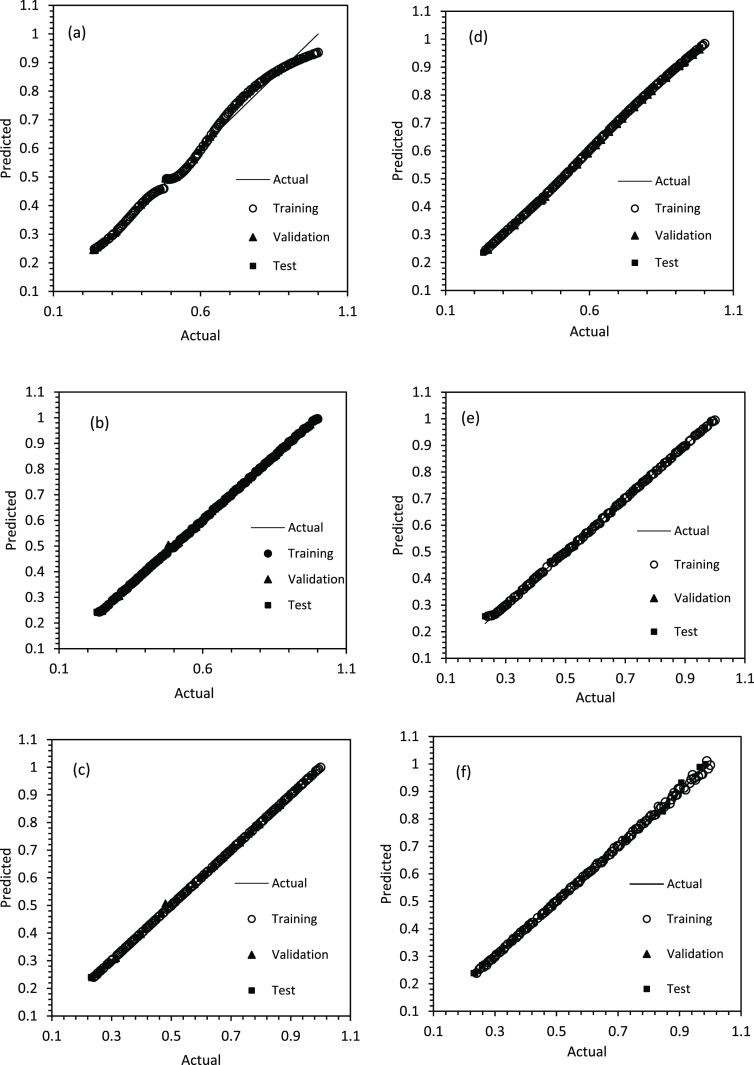
(a) ANN model for SS1 and SS2, (b) random forest model for SS1 and SS2, (c) XG-Boost model for SS1 and SS2, (d) ANN model for SS3, (e) random forest model for SS3, and (f) XG-Boost model for SS3.

The performance of both developed SS1 and SS2, based ANN, RF, and XG-Boost models is shown in Figure 11a,b,c, respectively. Therefore, the whole range of data is visible. The performance of SS3 is presented in Figure 11d,e,f. Figure 11 demonstrates the models output based on train, validation, and test data sets for all soft sensors. It can be seen from Figure 11 that all models are well-suited for predicting the surface area. For SS1 and SS2, the XG-Boost and RF models exhibited better performance (see Figure 11a,b,c). For SS1, XG-Boost and RF models achieved *R*-squared values of 0.9973 and 0.9951, with mean absolute errors of 0.0019 and 0.0032, respectively. In the case of SS2, the XG-Boost and RF models demonstrated remarkable *R*-squared values of 0.9998 and 0.9995, along with mean absolute errors of 0.0009 and 0.0023, respectively. For SS3, all three models exhibited excellent performance: ANN (*R*-square: 0.9998, MAE: 0.0022), RF (*R*-square: 0.9988, MAE: 0.0052), and XG-Boost (*R*-square: 0.9984, MAE: 0.0060).

In terms of time for training and estimation, all models required less than 1.85 s. The maximum iterations or maximum number of epochs needed for training in ANN models was 200. This number for the RF model in the SS1 and SS2 was 200 and in SS3 was 1000 and for XG-Boost in SS1 and SS2 was 1500 and in SS3 was 6000.

Each model has its own unique characteristics and considerations that can impact their training process and performance. XG-Boost is an implementation of gradient boosting, which is an ensemble method that builds models sequentially. Each subsequent model focuses on reducing the errors made by the previous models, leading to an improved overall performance. This model performs tree pruning during the construction of individual decision trees, removing tree branches that do not contribute significantly to the model’s performance. This helps to simplify the model and reduce the training time and overfitting.

**Figure 12:**
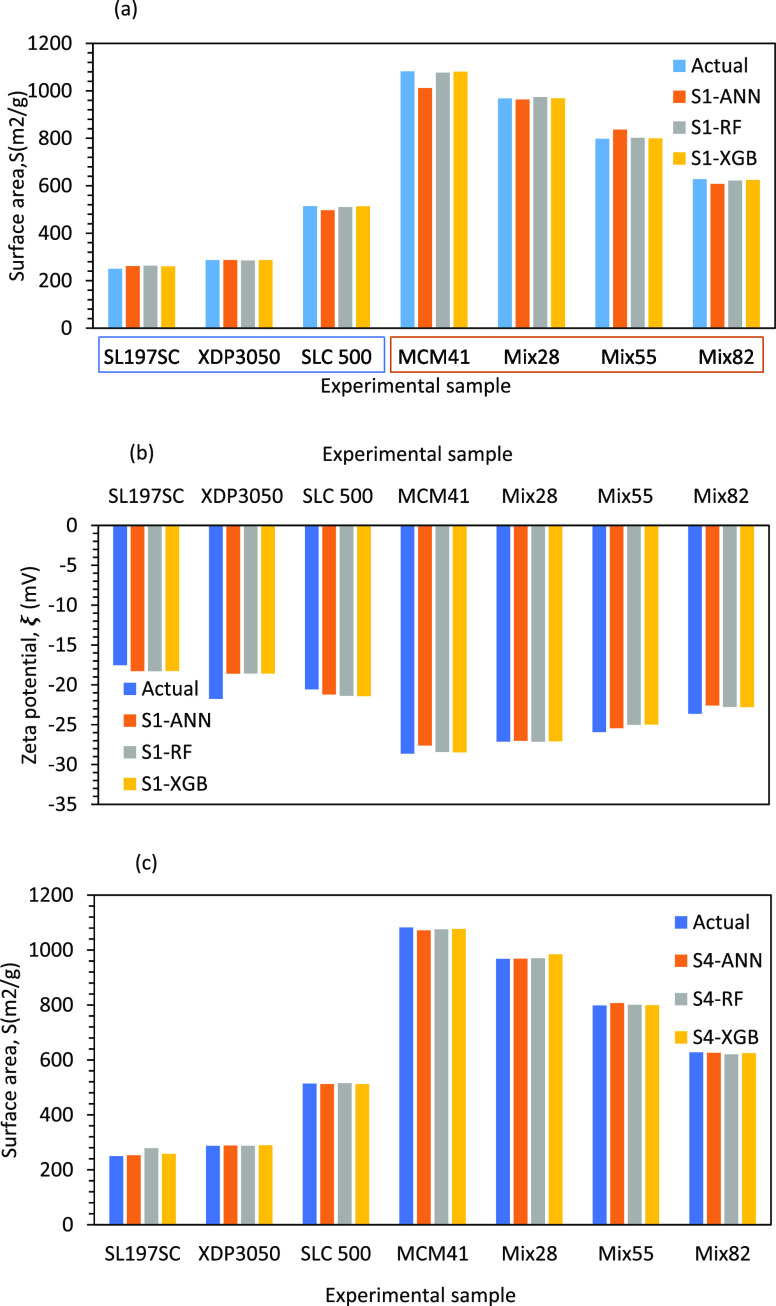
(a) Combined SS1 and SS2 for surface area as output. (b) Calculated zeta potential from area estimated with SS1 and SS2; (c) output of SS3 for surface area.

The developed soft sensors SS1 and SS2 were used for estimating the surface area for the seven experimental samples. The comparison of estimated values using SS1 and SS2 with experimental data is shown in Figure 12a. It can be seen that the soft sensors were able to estimate surface areas from the conductivity profiles quite well. The comparison of zeta potential calculated using area values estimated by SS1 and SS2 and eq 20 is shown in Figure 12b. It can be seen that the predicted zeta potential values also show good agreement with the experimental data. The comparison of area values predicted by SS3 with experimental data is shown in Figure 12c. Here again, SS3 was able to predict the values of surface area for the whole range using a single soft sensor.

In this study, the soft sensors for surface area prediction were developed using a set of conductivity profiles obtained via experimental measurements and a phenomenological physical adsorption model. The predicted surface area was utilized for the zeta potential estimation based on the proposed equation in this work. The performance of these soft sensors for entire data set showcased accurate predictions, indicating their potential for applications requiring quick estimations of the surface area of porous materials. The developed approach and method reduce overall measurement time for surface area from several hours to few minutes. The successful demonstration of these soft sensors in this work opens up opportunities for their utilization in a broader range of applications including near-online measurements for a wide range of porous materials.

## 5 Summary and Conclusions

This work presents an innovative and efficient approach and method for estimating the surface area of porous materials. The method uses measurement of conductivity during adsorption of conductive dye on porous materials and an ML-based soft sensor. Experiments were carried out to measure the conductivity profiles during dye adsorption for a range of commercial silica samples. A phenomenological model was developed to simulate dye adsorption on porous particles using two fitted parameters–surface area and a parameter of adsorption isotherm, *P*_1_. It was observed that the parameter *P*_1_ was correlated with the surface area and exhibited a parabolic-type relationship with surface area. Zeta potential was found to be linearly related with the surface area. Considering this relationship between the zeta potential and area, it was decided to develop a soft sensor for the surface area. Experimental data and simulated conductivity profiles obtained using a phenomenological model were used to develop soft sensors based on ANN, RF, and XG-Boost formalisms. Two different soft sensors (SS1 and SS2) were developed to cover the entire range of surface areas. Hyperparameter tuning was conducted to optimize the models. Both the soft sensors performed very well with *R*-squared greater than 0.99 on test data. The current approach completed the estimation of surface area within 15 min and achieved excellent accuracy (within 2.3% error). The zeta potential calculated from the estimated surface area also showed excellent agreement with the experimental data. For avoiding the use of two different soft sensors for covering the considered range of surface areas, a third soft sensor (SS3) was developed which used the value of zeta potential as an input. This soft sensor was able to predict the surface area over the entire range (from 250 to 1100 m^2^/g) with excellent accuracy. The developed approach, methods, and soft sensors will be very useful for researchers and engineers relying on the time-consuming BET technique to characterize the surface area of porous materials. The work opens up new opportunities for controlling the continuous synthesis of porous materials.

## Supporting Information Available

Calibration graph; normalized conductivity profiles with error bars; optimization of concentration for zeta potential measurement; result of BET analysis; surface area calculation for mixed samples; particle pore size distribution; adsorption modeling; and zeta potential modeling (PDF)

## Nomenclature

*S̅*: Surface of silica particles per volume of solution, m^–1^
*C*_D_: Concentration of dye in liquid, kg/m^3^
*C*_Ds_: Equilibrium concentration of dye in liquid, kg/m^3^
*S*: Surface area per mass of adsorbent, m^2^/kg
*m*_f_: mass of solid in slurry, kg
*y*_Ds_: Mass fraction of dye adsorbed on solid, kg/kg
α_s_: Volume fraction of solids
ρ_s_: Density of solids, kg/m^3^
*P*_1_: Parameter of adsorption isotherm, m^3^/kg
*P*_2_: Parameter of adsorption isotherm, m^3^/kg
*V*: Volume of solution, m^3^
*q*_F_: Feed rate of silica-containing slurry, m^3^/s
α_sF_: Volume fraction of solids in the feed slurry
*k*_SL_: Mass transfer coefficient, m/s
*t*_f_: Work time of pump, s
ζ: Zeta potential, mV
σ_eff_: Effective charge density
σ: Surface charge density
*T*: Temperature
ψ_0_: Surface electrostatic potential
ϵ_0_: Permittivity of vacuum or free space
ϵ_r_: Relative permittivity or dielectric constant
*N*: Avogadro constant
*k*_B_: Boltzmann’s constant
*N*_s_: Number of samples in training data set
*N*_i_: Number of input neurons
*N*_o_: Number of output neurons
*N*_h_: Number of hidden layers

## Subscripts

0: Initial (at *t* = 0)
