# Supplementary material for: Novel Machine Learning-Based Method for Estimation of the Surface Area of Porous Silica Particles
Source: Ind Eng Chem Res. 2023 Oct 27;62(44):18810–21. doi: 10.1021/acs.iecr.3c02785 (PMC10636746; doi:10.1021/acs.iecr.3c02785)
Supplement: Supplementary file 1 — ie3c02785_si_001.pdf [file ie3c02785_si_001.pdf]

Supplementary Information

A Novel Machine Learning-based Method for Estimation of Surface Area of Porous Silica Particles

Roja P. Moghadam, Chinmay A. Shukla and Vivek V. Ranade*
Multiphase Reactors and Process Intensification Group
Bernal Institute, University of Limerick, V94T9PX, Ireland
* Corresponding author: Vivek.Ranade@ul.ie

Contents

1. Calibration graph ... 2
2. Error bar on conductivity profiles ... 2
3. Optimazation of concentration for zeta potential measurement ... 3
4. Result of BET analysis ... 3
5. Surface area calculation for mixed samples ... 4
6. Particle pore size distribution ... 4
7. Adsorption modelling ... 5
8. Zeta potential modelling ... 8
9. References ... 11

1. Calibration graph

Figure S1 shows a linear relation between conductivity and concentration of dye, based on calibration of dye concentration.

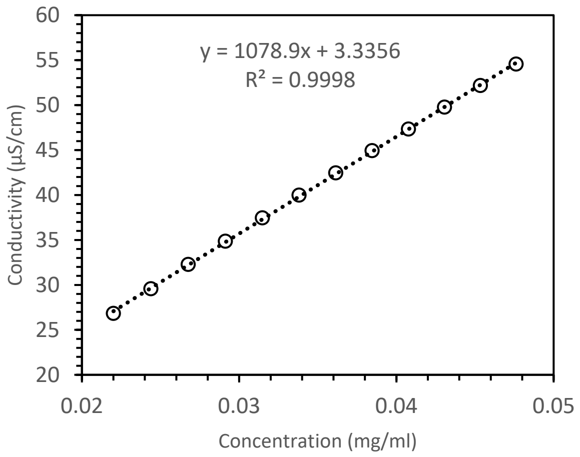

Figure S1. Conductivity versus concentration

2. Error bar on conductivity profiles

Dye adsorption experiments were repeated for three times and error bars for conductivity profiles of various silica particles via experimental repetition were provided in Figure S2.

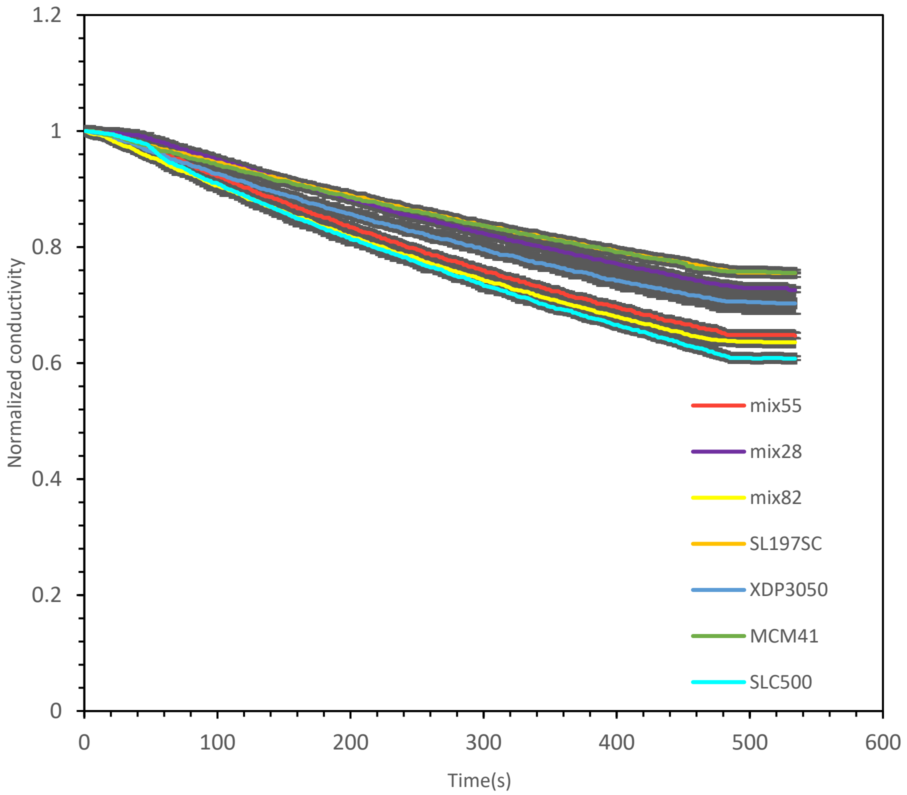

Figure S2. Error bars for normalized conductivity profiles of various silica particles via experimental repetition

3. Optimazation of concentration for zeta potential measurement

Zeta potential measurement for Merk sample with different concentrations (0.833, 1.66, 2.5, 3.33, 4 mg/mL) with three repetitions. According to Figure S3, to concentrations 1.66 mg/mL and 2.5 mg/mL demonstrated lower standard deviation:

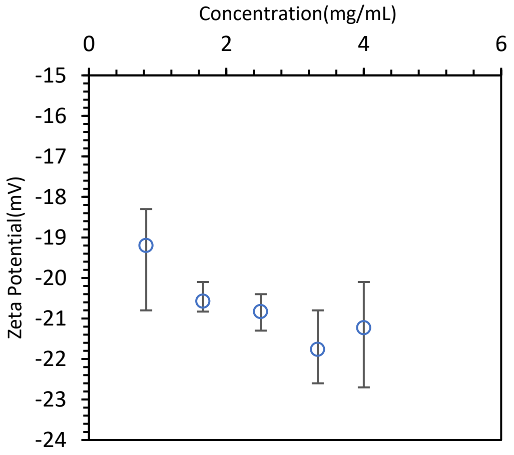

Figure S3. Zeta potential measurement for Merk sample with different concentrations

4. Result of BET analysis

The isotherm adsorption and desorption of N2 in BET analysis for four silica samples, as displayed in Table 1, was shown in Figure S4.

These reversible isotherm plots are the forms of isotherm obtained with the micropores and mesopores adsorbent and represent unrestricted monolayer-multilayer adsorption. The beginning of the almost linear middle section of the isotherm often indicates the stage at which monolayer coverage is complete and multilayer adsorption about to begin. The hysteresis is usually attributed to the thermodynamic or network effects or the combination of these two effects. The high steepness of the isotherms indicates the relatively high pore size uniformity and facile pore connectivity, and the less steepness presents narrow slit-like pores, particles with internal voids of irregular shape and broad size distribution, hollow spheres with walls composed of ordered mesoporous silica. Also, the extended double-sided arrow shows the larger surface area[1-3]

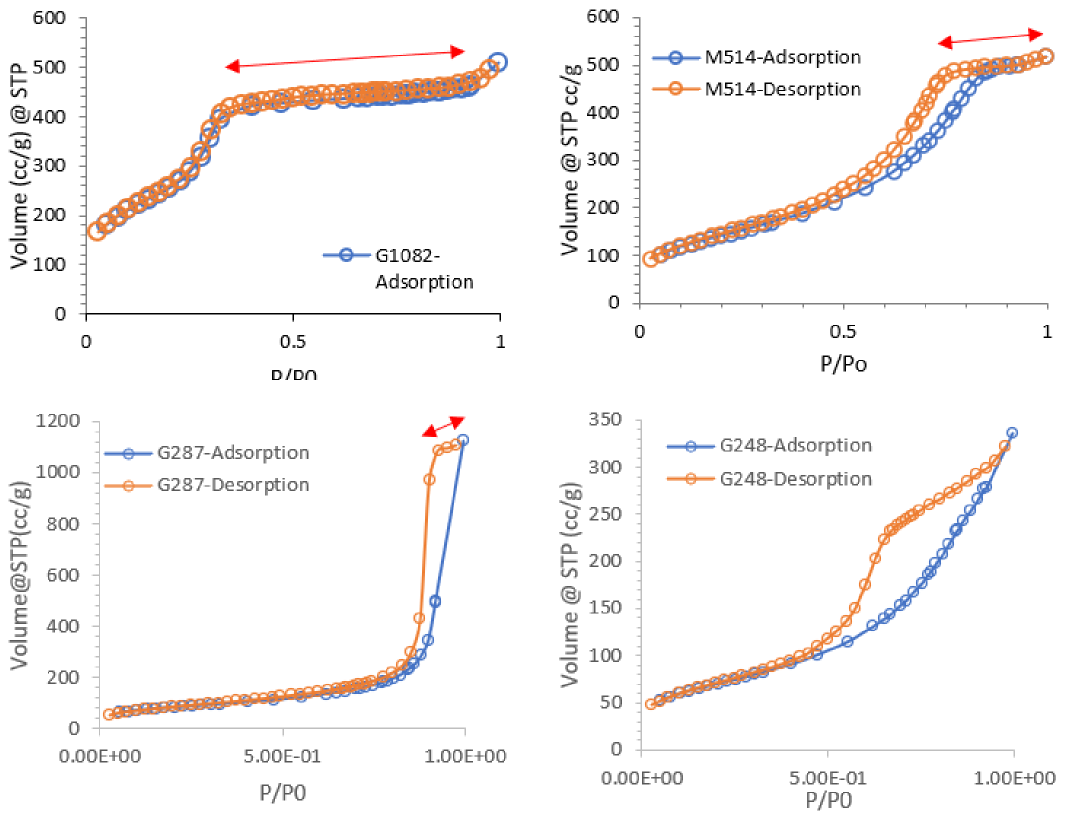

Figure S4. Isotherm adsorption and desorption of N_2 for silica samples with different surface area in BET test.

5. Surface area calculation for mixed samples

Calculation of surface area for a mix of particles in different sizes:

i=1, 2, 3, …, n

S: surface area, (m^2/g)

m: weight, (g)

$$S_{total} = \frac{\sum_{i=1}^{n} S_i \times m_i}{m_{total}}$$

Example:

$$S_1 = 1082,\ S_2 = 514\ \ m_1 = 0.04,\ m_2 = 0.01\ \ =>\ \ S_{total} = \frac{1082\times0.04+514\times0.01}{0.05} = 968.4$$

6. Particle pore size distribution

We have used the BJH method [4] to measure pore size distributions of all the silica particles used in this work. The results are shown here in Figure S5:

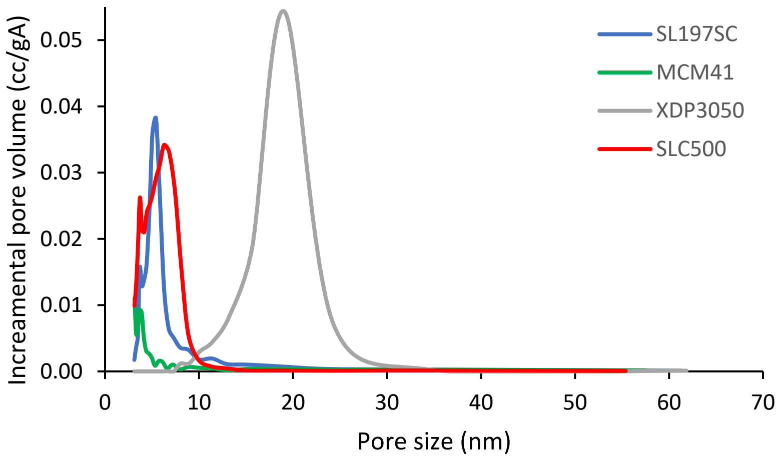

Figure S5: Pore size distribution for silica samples.

7. Adsorption modelling

$$\frac{d(VC_D)}{dt} = -V\, k_{SL}\bar{S}\,(C_D - C_{Ds}) \tag{1}$$

$$\frac{dV}{dt} = q \tag{2}$$

$$\frac{d(V\alpha_s)}{dt} = q\ \alpha_{sF}\ q \tag{3}$$

$$q = q_F \qquad t \le t_F \tag{4}$$

$$q = 0 \qquad t > t_F \tag{5}$$

Thus, we get:

$$V = V_0 + q_F t \qquad t < t_F \qquad and \quad V = V_0 + q_F t_F \qquad t > t_F \tag{6}$$

$$V\frac{d\alpha_s}{dt} = q_F\,(\alpha_{sF} - \alpha_s) \qquad t \le t_F \tag{7}$$

$$\alpha_s = \alpha_{sF}\left(1 - e^{-q_F t/V}\right) \qquad t \le t_F \tag{8}$$

$$\alpha_s = \alpha_{sF}\left(1 - e^{-q_F t_F/V}\right) \qquad t > t_F \tag{9}$$

Following the Langmuir isotherm, the mass fraction of dye adsorbed on the solid is:

$$y_{Ds} = \frac{P_1 C_{Ds}}{1 + P_2 C_{Ds}} \tag{10}$$

Overall mass balance relates y_{Ds} with C_D as:

$$\frac{d}{dt}(VC_D + V\alpha_s\,\rho_s\,y_{Ds}) = 0 \tag{11}$$

$$VC_D + V\alpha_s\,\rho_s\,y_{Ds} = VC_{D0} \tag{12}$$

$$y_{Ds} = \frac{C_{D0} - C_D}{\alpha_s\,\rho_s} \tag{13}$$

$$\frac{1}{P_1 C_{Ds}} + \frac{P_2}{P_1} = \frac{\alpha_s\,\rho_s}{C_{D0} - C_D} \tag{14}$$

$$C_{Ds} = \frac{1}{P_1\left(\frac{\alpha_s\,\rho_s}{C_{D0} - C_D} - \frac{P_2}{P_1}\right)} \tag{15}$$

For low concentration of dye, $P_2\ \rightarrow 0$

$$C_{Ds} = \frac{C_{D0} - C_D}{P_1\alpha_s\,\rho_s} \tag{16}$$

The dye mass balance therefore becomes:

$$q_F C_D + V\frac{dC_D}{dt} = -V\,k_{SL}\bar{S}\,\left(C_D - \frac{C_{D0} - C_D}{P_1\alpha_s\,\rho_s}\right) \quad t \leq t_F \tag{17}$$

$$q_F C_D + (V_0 + q_F t)\,\frac{dC_D}{dt} = -(V_0 + q_F t)\,k_{SL}\bar{S}\,\left(C_D - \frac{C_{D0} - C_D}{P_1\alpha_s\,\rho_s}\right) \quad t \leq t_F \tag{18}$$

$$\frac{dC_D}{dt} = -\,k_{SL}\bar{S}\,\left(C_D - \frac{C_{D0} - C_D}{P_1\alpha_s\,\rho_s}\right) - \frac{q_F C_D}{(V_0 + q_F t)} \quad t \leq t_F \tag{19}$$

$$\frac{dC_D}{dt} = -\,k_{SL}\bar{S}C_D\,\left(1 + \frac{1}{P_1\alpha_s\,\rho_s} + \frac{q_F}{k_{SL}\bar{S}(V_0 + q_F t)}\right) + \frac{k_{SL}\bar{S}C_{D0}}{P_1\alpha_s\,\rho_s} \quad t \leq t_F \tag{20}$$

$$(V_0 + q_F t_F)\frac{dC_D}{dt} = -(V_0 + q_F t_F)\,k_{SL}\bar{S}\,\left(C_D - \frac{C_{D0} - C_D}{P_1\alpha_s\,\rho_s}\right) \quad t > t_F \tag{21}$$

$$\frac{dC_D}{dt} = -\,k_{SL}\bar{S}\,C_D\left(1 + \frac{1}{P_1\alpha_s\,\rho_s}\right) + \frac{k_{SL}\bar{S}\,C_{D0}}{P_1\alpha_s\,\rho_s} \quad t > t_F \tag{22}$$

For $t \leq t_F$:

$$\frac{dC_D}{dt} = -\,k_{SL}\bar{S}C_D\,\left(1 + \frac{1}{P_1\alpha_s\,\rho_s} + \frac{q_F}{k_{SL}\bar{S}(V_0 + q_F t)}\right) + \frac{k_{SL}\bar{S}C_{D0}}{P_1\alpha_s\,\rho_s} \quad t \leq t_F \tag{23}$$

$$\frac{dC_D}{dt} = a - b\,C_D - \frac{C_D}{t + \tau} \tag{24}$$

$$a = \frac{k_{SL}\bar{S}\,C_{D0}}{P_1\alpha_s\,\rho_s} \tag{25}$$

$$b = k_{SL}\bar{S}\left(1 + \frac{1}{P_1 \alpha_s \rho_s}\right) \tag{26}$$

$$\tau = \frac{V_0}{q_F} \tag{27}$$

We have:

$$\alpha_s = \frac{\alpha_{sF} q_F t}{(V_0 + q_F t\,)} \qquad , \qquad \bar{S} = S\, m_f t \Big/ (V_0 + q_F t\,) \qquad t \le t_F \tag{28}$$

$$a = \frac{k_{SL} S\, m_f C_{D0}}{P_1 \alpha_{sF} q_F \rho_s} \quad t \le t_F \tag{29}$$

$$b = \frac{k_{SL} S\, m_f q_F t}{(t+\tau)} + \frac{k_{SL} S\, m_f}{P_1 \alpha_{sF} q_F \rho_s} \quad t \le t_F \tag{30}$$

$$b = \frac{k_{SL} S\, m_f q_F t}{(t+\tau)} + \frac{a}{C_{D0}} \quad t \le t_F \tag{31}$$

$$h = k_{SL} S\, m_f q_F \tag{32}$$

$$b = \frac{ht}{(t+\tau)} + \frac{a}{C_{D0}} \quad t \le t_F \tag{33}$$

$$\frac{dC_D}{dt} = a - \left(\frac{ht}{(t+\tau)} + \frac{a}{C_{D0}}\right) C_D - \frac{C_D}{t+\tau} \quad t \le t_F \tag{34}$$

ODE solution By MATLAB:

```
>> dsolve('Dy-a+((h*x/(x+T))+(a/x0))*y+(y/(x+T))=0','x')

ans =

C1*exp(-(x*(a + h*x0))/x0)*(T + x)^(T*h - 1) + (a*x0*exp(-(x*(a + h*x0))/x0)*exp(- T*h - (T*a)/x0)*(T + x)^(T*h - 1)*(-x0/(a + h*x0))^(1 - T*h)*igamma(2 - T*h, -((T + x)*(a + h*x0))/x0))/(a + h*x0)
```

Therefore :

$$C_D = C_1\,(t+\tau)^{(h\tau-1)} e^{-(hC_{D0}+a)/C_{D0})t} + \Gamma\left(2 - h\tau, \frac{-(hC_{D0}+a)(t+\tau)}{C_{D0}}\right) \frac{aC_{D0}\,(-C_{D0}/(hC_{D0}+a))^{(1-h\tau)}\; e^{-(\frac{\tau a}{C_{D0}}+h\tau)}}{(hC_{D0}+a)} (t+\tau)^{(h\tau-1)} e^{-(hC_{D0}+a)/C_{D0})t} \;\; t \le t_F \tag{35}$$

The following term is constant:

$$C_2 = \Gamma\left(2 - h\tau, \frac{(hC_{D0} + a)(t+\tau)}{C_{D0}}\right) \frac{aC_{D0}\,(-C_{D0}/(hC_{D0}+a))^{(1-h\tau)}\ e^{-(\frac{\tau a}{C_{D0}}+h\tau)}}{(hC_{D0}+a)} \tag{36}$$

$$C_D = C_1\,(t+\tau)^{(h\tau-1)} e^{-(hC_{D0}+a)/C_{D0})t} + C_2\,(t+\tau)^{(h\tau-1)} e^{-(hC_{D0}+a)/C_{D0})t} \tag{37}$$

$$C_0 = C_1 + C_2 \tag{38}$$

Final ODE solution:

$$C_D = C_0\,(t+\tau)^{(h\tau-1)} e^{-(hC_{D0}+a)/C_{D0})t} \quad t \le t_F \tag{39}$$

The final ODE solution was applied for prediction of C_D.

$$(hC_{D0} + a)/C_{D0}) = k_{SL}S\,m_f(q_F + \frac{1}{P_1\alpha_{sF}q_F\rho_S}) \quad t \le t_F \tag{40}$$

$$C_D = C_0(t+\tau)^{(k_{SL}S\,m_f q_F\tau - 1)}\ e^{-k_{SL}S\,m_f(q_F + \frac{1}{P_1\alpha_{sF}q_F\rho_S})t} \quad t \le t_F \tag{41}$$

Initial condition: $C_D(t=0) \ \rightarrow\ C_{D0}$

$$C_D = C_{D0}(t+\tau)^{(k_{SL}S\,m_f q_F\tau - 1)}\ \frac{e^{-k_{SL}S\,m_f(q_F + \frac{1}{P_1\alpha_{sF}q_F\rho_S})t}}{\tau^{(k_{SL}S\,m_f q_F\tau - 1)}} \quad t \le t_F \tag{43}$$

For $t > t_F$:

$$q = 0\ ,\quad \frac{dV}{dt} = 0 \quad ,\quad C_D = C_{Ds} \quad t > t_F \tag{44}$$

$$\frac{dC_D}{dt} = 0 \qquad t > t_F \tag{45}$$

$$C_D = C_{Dat\ t=t_F} \tag{46}$$

8. Zeta potential modelling

The surface charge density (σ) is calculated using the following equation[5]:

$$\sigma = F\frac{C_M V}{m\,S} \tag{47}$$

where C_M is the acidic or basic reagent concentration, *V* is the reagent volume, *m* is the weight of powder, and *S* is the BET specific area.

The Gouy-Chapman theory establishes a direct relationship between zeta potential and the "effective charge density" based on the sum of charge density on a flat surface and the surrounding ions enclosed by the slipping plane[5]. The resulting nonlinear Poisson−Boltzmann equation has an exact solution in the 1-dimensional case, which is known as the Gouy−Chapman equation for a flat-charged surface:

$$\sigma = \sqrt{8\, c\, N\, \epsilon_r\, \epsilon_0\, K_B\, T}\, \sinh\,(\frac{e\psi_0}{2\, K_B T}) \qquad (48)$$

where *c* stands for the ion concentration, *N* is the Avogadro constant, k_B is Boltzmann's constant, ϵ_r is relative permittivity or dielectric constant, ϵ_0 is permittivity of vacuum or free space, *T* is the temperature, and σ and ψ_0 are the surface charge density and surface electrostatic potential, respectively. While the Gouy−Chapman equation describes the relation between ψ_0 and σ, the very same relation exists between zeta potential *(ξ)* and the effective charge density *(σ_{eff})* described earlier[6]:

$$\sigma_{eff} = \sqrt{8\, c\, N\, \epsilon_r\, \epsilon_0\, K_B\, T}\, \sinh\,(\frac{e\xi}{2\, K_B T}) \qquad (49)$$

Based on the above equations, there is the following relation between surface area and zeta potential:

$$\frac{C_3}{S} = C_2 \sinh\,(C_1 \xi) \qquad (50)$$

$$\frac{1}{S} = C_4 \sinh\,(C_1 \xi) \qquad (51)$$

$$S = \frac{1}{C_4 \sinh\,(C_1 \xi)} \qquad (52)$$

$$\sinh(x) = \frac{e^x - e^{-x}}{2}$$

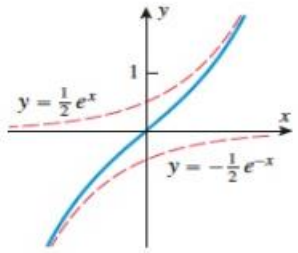

$$\frac{1}{\sinh(x)} = \operatorname{csch}\,(x)$$

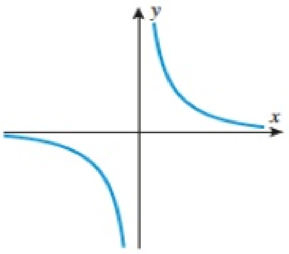

y = csch x

In our case, surface area is positive. Therefore y>0 in csch(x) is acceptable. Hence, by applying the mapping method, the final form of graph will be like this:

(y: Surface area, x: zeta potential)

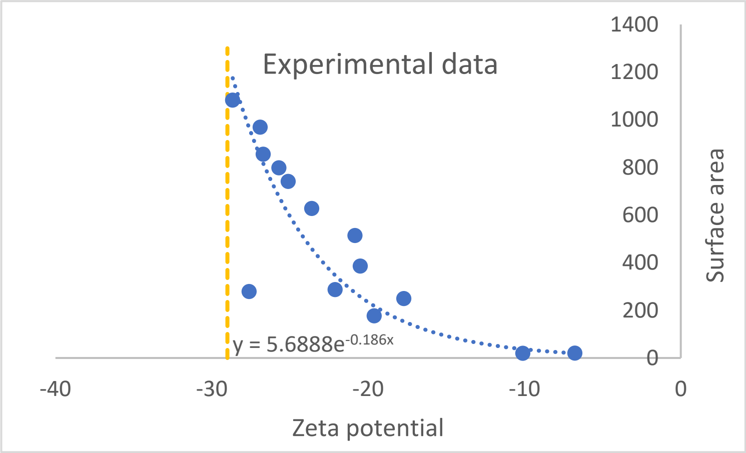

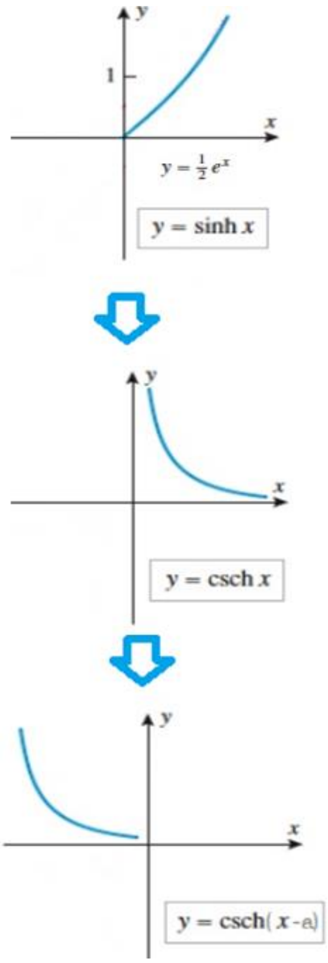

With considering the above description, the final form of equation will be:

$$S = \frac{1}{C_4 \sinh\,(C_1 \xi)} \quad (53)$$

$$S = C_5\; e^{[-C_1(\xi - \xi_0)]} \quad (54)$$

Therefore, zeta potential versus surface area will be based on the natural log, or ln function. But about this case, With considering the Eq. (54), For small values of $C_1(\xi - \xi_0)$, the final form of equation can be linear:

$$\boldsymbol{S = C_5\,[1 + C_1(\xi - \xi_0)] = C_5 + C_5 C_1(\xi - \xi_0)} \quad (55)$$

The unknown parameters were calculated by back-fitting and based on experimental data.

$$\xi = -21.5 - \frac{(S - 520)}{80.32} \quad (56)$$

9. References

(1) Kruk, M.; Jaroniec, M. Gas Adsorption Characterization of Ordered Organic−Inorganic Nanocomposite Materials. *Chemistry of Materials* **2001**, *13* (10), 3169–3183. DOI: https://doi.org/10.1021/cm0101069.

(2) Sing, K. S. Adsorption methods for the characterization of porous materials. *Advances in colloid and interface science* **1998**, *76*, 3-11. DOI: https://doi.org/10.1016/S0001-8686(98)00038-4.

(3) Groen, J. C.; Peffer, L. A. A.; Pérez-Ramírez, J. Pore Size Determination in Modified Micro- and Mesoporous Materials. Pitfalls and Limitations in Gas Adsorption Data Analysis. *Microporous and Mesoporous Mater.* **2003**, *60* (1-3), 1-17. DOI: https://doi.org/10.1016/S1387-1811(03)00339-1.

(4) Barrett, E. P.; Joyner, L. G.; Halenda, P. P. The Determination of Pore Volume and Area Distributions in Porous Substances. I. Computations from Nitrogen Isotherms. *The Volume and Area Distributions in Porous Substances* **1951**, (73), 373-380. DOI: https://doi.org/10.1021/ja01151a046.

(5) Pochapski, D. J.; Santos, C. C. d.; Leite, G. W.; Sandra Helena Pulcinelli; Santilli, a. C. V. Zeta Potential and Colloidal Stability Predictions for Inorganic Nanoparticle Dispersions: Effects of Experimental Conditions and Electrokinetic Models on the Interpretation of Results. *Langmuir* **2021**, *37*, 13379-13389. DOI: https://doi.org/10.1021/acs.langmuir.1c02056.

(6) Ge, Z.; Wang, Y. Estimation of Nanodiamond Surface Charge Density from Zeta Potential and Molecular Dynamics Simulations. *Journal of Physical Chemistry B* **2016**, *121*, 3394-3402. DOI: https://doi.org/10.1021/acs.jpcb.6b08589.
